# Patient‐reported symptom burden and impact on daily activities in chronic graft‐versus‐host disease

**DOI:** 10.1002/cam4.5209

**Published:** 2022-11-16

**Authors:** Jingbo Yu, Betty K. Hamilton, James Turnbull, Susan K. Stewart, Alla Vernaya, Valkal Bhatt, Oren Meyers, John Galvin

**Affiliations:** ^1^ Incyte Corporation Wilmington Delaware USA; ^2^ Cleveland Clinic Cleveland Ohio USA; ^3^ Patient Centered Endpoints, IQVIA New York New York USA; ^4^ Blood & Marrow Transplant Information Network Highland Park Illinois USA

**Keywords:** hematological cancer, myeloproliferative disorders, QoL, quality of life

## Abstract

**Background:**

Chronic graft‐versus‐host disease (GVHD) is a potentially life‐threatening complication of allogeneic hematopoietic stem cell transplantation (HSCT) treatment for hematologic malignancies. There are limited patient‐reported data concerning symptom burden and effects on activities of daily living (ADL).

**Methods:**

The cross‐sectional *Living With Chronic GVHD Patient Survey* was administered online in the United States (May–August 2020) to participants aged ≥18 years who underwent allogeneic HSCT, were diagnosed with chronic GVHD by a healthcare provider, and self‐reported active chronic GVHD (within past 5 years). Information on patient demographics, disease characteristics, symptom burden, and ability to perform ADL was collected. Symptom burden was assessed using the validated Lee Symptom Scale (range from 0–100 with higher scores indicating greater burden). All data were summarized using descriptive statistics; no formal statistical comparisons were conducted.

**Results:**

Out of 580 participants who entered the survey screener, 165 participants (28.4%) across 33 states fulfilled all study eligibility criteria, completed the entire survey, and were included (age: mean [SD], 53.7 (13.8) years; median [range], 57.0 [18–78] years; female, *n* = 105 [63.6%]; White, *n* = 137 [83.0%]). Respondents described their chronic GVHD severity primarily as moderate (*n* = 54 [32.7%]) or severe (*n* = 102 [61.8%]) at the time when symptoms were at their worst. One‐third of respondents (33.9%) indicated that their chronic GVHD symptoms were at their worst for ≥1 year in duration. Mean (SD; range) Lee Symptom Scale score was 44.8 (19.4; 2–100); 44% of respondents considered “dry eye” the most burdensome symptom. Almost half of respondents (*n* = 73 [44.2%]) rated their overall quality of life (QoL) as poor. Participants reported a detrimental impact of symptoms on ADL, including basic activities (eg, eating, personal hygiene, dressing).

**Conclusions:**

Survey respondents self‐reported high chronic GVHD symptom burden and felt that their symptoms severely interfered with physical function and ADL. Effective strategies to mitigate chronic GVHD symptoms are needed to improve QoL among HSCT survivors.

## Graphical abstract

Patients with chronic GVHD experience burdensome symptoms that impair their ability to perform daily activities and reduce their quality of life. There remains an unmet need for effective therapies that alleviate symptoms of chronic GVHD and improve patients’ physical and social function.Respondent‐reported condition when chronic GVHD symptoms were at their worst.

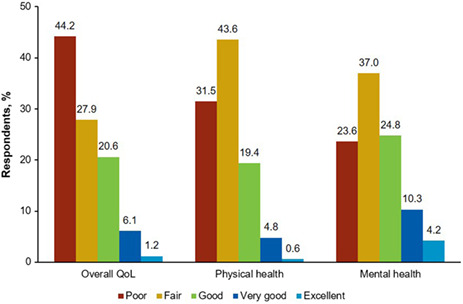

## 1 INTRODUCTION

Allogeneic hematopoietic stem cell transplantation (HSCT) is the only potentially curative treatment for several hematologic disorders and is steadily increasing in use, with approximately 9500 procedures performed in 2019 in the United States alone.^1^, ^2^, ^3^, ^4^ Chronic graft‐versus‐host disease (GVHD) is a late effect of allogeneic HSCT that occurs in approximately 30% to 50% of HSCT recipients.^5^, ^6^, ^7^, ^8^, ^9^ Among long‐term HSCT survivors (ie, ≥2 years), chronic GVHD is a leading cause of nonrelapse mortality.^10^, ^11^ Furthermore, development of chronic GVHD is associated with reduced health‐related quality of life (HRQoL) and impaired physical functioning after HSCT.^12^, ^13^, ^14^, ^15^, ^16^, ^17^, ^18^

Clinical manifestations of chronic GVHD include multi‐organ pathology with common involvement of the skin, mouth, eyes, joints, genitals, gastrointestinal tract, liver, and lungs,^19^ and previous reports suggest that HRQoL and functional performance status of patients with chronic GVHD may be affected by organ involvement and severity of organ‐specific symptoms.^20^ In a previous cross‐sectional natural history study of patients with chronic GVHD, National Institutes of Health (NIH) global severity scores were negatively associated with several functional and HRQoL outcome measures, with the presence of sclerotic skin, joints/fascia manifestations, or lung involvement having the greatest detrimental effect.^21^ One of the HRQoL tools used in the study was the Lee Symptom Scale (LSS), a validated 30‐item scale with 7 subscales designed to capture chronic GVHD‐specific symptom burden.^22^ LSS scores were associated with individual organ NIH severity scores across most organs, with the most significant correlation observed for gastrointestinal involvement.^21^ Additional reports have shown that chronic GVHD symptom burden directly affects functional performance status^17^ and that higher chronic GVHD symptom burden is associated with self‐reported depression and anxiety.^23^ Notably, the LSS is the only validated instrument designed for use specifically in patients with chronic GVHD.

Although several prior studies have evaluated HRQoL and physical function among patients with chronic GVHD symptoms, there are limited data available regarding patient‐reported impact of their symptoms on the ability to carry out daily activities. Therefore, contemporaneous, comprehensive analyses examining chronic GVHD symptoms and their impact on activities of daily living from the patient's perspective are needed to better understand the extent of physical burden faced by patients in their everyday lives. The objective of this analysis was to measure the symptom burden of chronic GVHD and explore patients' perceptions of the effects of symptoms on their daily activities using direct patient input from the *Living With Chronic GVHD Patient Survey*.

## 2 METHODS

### 2.1 Study design

The *Living With Chronic GVHD Patient Survey* was a cross‐sectional, noninterventional online survey of participants aged ≥18 years who live in the United States, underwent allogeneic HSCT, received a chronic GVHD diagnosis from a healthcare provider, and self‐reported active chronic GVHD (within the past 5 years). The survey was programmed and piloted using 3 patient interviews, after which the final version was administered in the United States from May to August 2020. Participants were recruited via email blasts, newsletters, and Facebook posts sent by chronic GVHD patient advocacy groups and consumer panels.

Upon clicking the survey link, patients entered a screener in which they were asked to provide consent and respond to 4 screening questions to determine survey eligibility. The survey had 74 items and took approximately 30 minutes to complete. Information on patient demographics, transplant date, chronic GVHD characteristics (date of diagnosis; specialists visited; disease duration; systemic treatments received; functional impact due to chronic GVHD at diagnosis and at its worst severity; and date, severity, and organ involvement), symptom burden when chronic GVHD was at its worst, and change in ability to perform regular daily activities was collected and used for this analysis. Symptom burden was assessed using the validated LSS (total score ranging from 0–100, with a higher score indicating more bothersome symptoms)^22^ and a separate genital and urinary subscale. The LSS is composed of 7 subdomains (eyes, energy, psychological, skin, mouth, nutrition, and lung) covering 30 individual symptoms; the genital and urinary subscale assesses 4 individual symptoms (Table S1). Respondents were asked to rate their overall QoL and physical and mental health at diagnosis and when chronic GVHD symptoms were at their worst, with the following choices: “excellent,” “very good,” “good,” “fair,” and “poor.” Activities of daily living were measured on a scale of 0 (“chronic GVHD had no effect on my daily activities”) to 10 (“chronic GVHD completely prevented me from doing my daily activities”). Basic activities of daily living were defined as the skills required to manage basic physical needs, whereas instrumental activities of daily living were those requiring more complex organizational and cognitive abilities. Participants received $25 honoraria payments from the vendor for completing the survey.

### 2.2 Ethical considerations

The study was conducted in accordance with the ethical principles embodied by the Declaration of Helsinki and Good Clinical Practice. The survey, including revisions based on the pilot in 3 participants, was reviewed and approved by the New England Institutional Review Board before implementation. All survey respondents provided informed consent via an online consent form before screening and survey completion.

### 2.3 Statistical analyses

Only respondents who completed the entire survey (ie, no missing responses) were included in the analysis. Survey responses were analyzed overall and stratified by respondent‐specified chronic GVHD severity (mild/moderate vs severe) and duration (<6 vs ≥6 months). For statistical analyses, scores pertaining to activities of daily living (reported by survey respondents on a scale of 0–10) were coded using positive integers from 1 (corresponding to a response of 0, “chronic GVHD had no effect on my daily activities”) to 11 (corresponding to a response of 10, “chronic GVHD completely prevented me from doing my daily activities”). Individual LSS items were scored based on a scale of 0 (not at all bothersome) to 4 (extremely bothersome) and linearly transformed to a 0 to 100 scale. All data processing, summarization, and analyses were performed in SAS version 9.4 (SAS Institute Inc., Cary, NC, USA). All data including frequencies, mean (SD), and median (range) were summarized using descriptive statistics. The study was descriptive in nature, and no formal analyses of associations between disease duration or severity and outcomes were conducted.

## 3 RESULTS

### 3.1 Respondents

**FIGURE 1:**
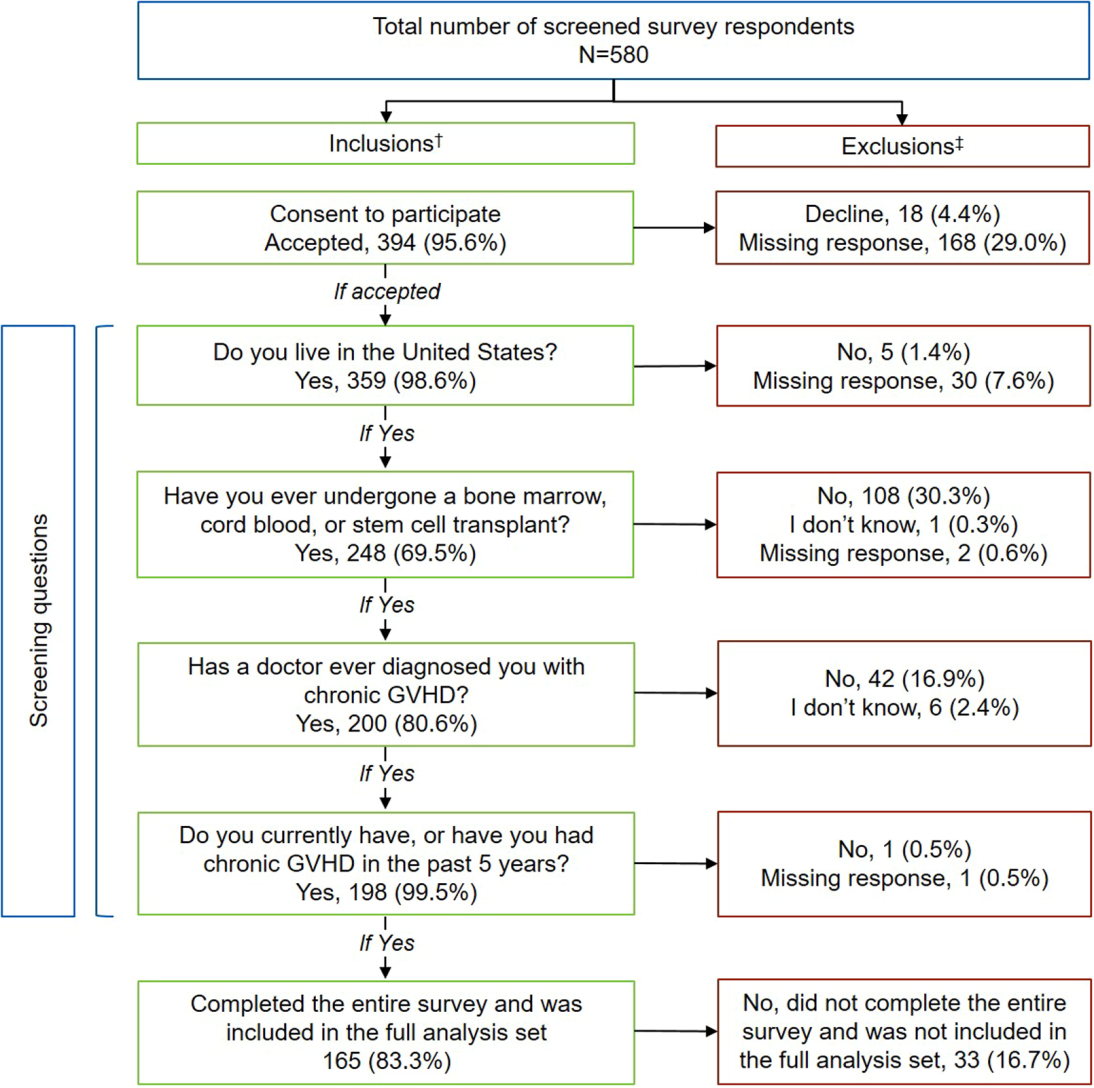
Respondent disposition. ^†^Percentages are out of the number of participants with a nonmissing response. ^‡^Percentages for exclusions (“decline” or “no”) are out of the number of participants with a nonmissing response.

Out of 580 initial survey respondents, the analysis set included 165 respondents (28.4%) from 33 states who fulfilled all eligibility criteria and completed the survey. The majority of included respondents were recruited from BMT InfoNet (*n* = 110); the remaining respondents were recruited from nbmtLINK (*n* = 22), Rare Patient Voice (*n* = 16), Be the Match (*n* = 6), Cowden Foundation (*n* = 6), and SurveyHealth (*n* = 5). Primary reasons for study exclusion among initial survey respondents included declined or missing consent (*n* = 186), not having received prior HSCT (*n* = 111 including unknown/missing response), and not having a diagnosis of chronic GVHD (*n* = 48 including unknown; Figure 1). The mean (SD) age was 53.7 (13.8) years (median, [range], 57.0 [18–78] years), 105 respondents (63.6%) were female, and 137 (83.0%) were White (Table 1). The median (range) time from chronic GVHD diagnosis to survey completion was 4.5 (0.1–36.7) years, with 155 respondents (93.9%) experiencing chronic GVHD symptoms at the time of the survey. More than half of patients (*n* = 95 [57.6%]) were diagnosed with acute GVHD before chronic GVHD; median (range) time from transplant to chronic GVHD diagnosis was 0.5 (0–3.6) years. Treatments that respondents reported receiving for chronic GVHD included corticosteroids (oral or intravenous; *n* = 140 [84.8%]), topical cream (*n* = 132 [80.0%]), other oral or intravenous treatment (*n* = 130 [78.8%]), ocular therapy (*n* = 102 [61.8%]), and extracorporeal photopheresis (*n* = 54 [32.7%]).

**TABLE 1:** Respondent demographics and clinical characteristics at the time of the *Living With Chronic GVHD Patient Survey*

Characteristic	Respondents with chronic GVHD (*N* = 165)
Age, years	
Median (range)	57.0 (18–78)
Mean (SD)	53.7 (13.8)
Sex, *n* (%)	
Female	105 (63.6)
Male	60 (36.4)
Ethnicity, *n* (%)	
White	137 (83.0)
Hispanic/Latino	11 (6.7)
Black/African American	7 (4.2)
Asian	5 (3.0)
Other	5 (3.0)
Diagnosis of acute GVHD before chronic GVHD, *n* (%)	
Yes	95 (57.6)
No	54 (32.7)
Unknown	16 (9.7)
Chronic GVHD duration, median (range), years	4.5 (0.1–36.7)
Chronic GVHD severity at diagnosis, *n* (%)	
Mild	37 (22.4)
Moderate	71 (43.0)
Severe	57 (34.5)
Treatments taken for chronic GVHD, *n* (%)	
Corticosteroids (oral or injection/infusion)	140 (84.8)
Topical cream	132 (80.0)
Other oral or injection treatment	130 (78.8)
Therapies for ocular GVHD	102 (61.8)
Extracorporeal photopheresis	54 (32.7)
None	1 (0.6)

Abbreviation: GVHD, graft‐versus‐host disease.

### 3.2 Symptom burden

**FIGURE 2:**
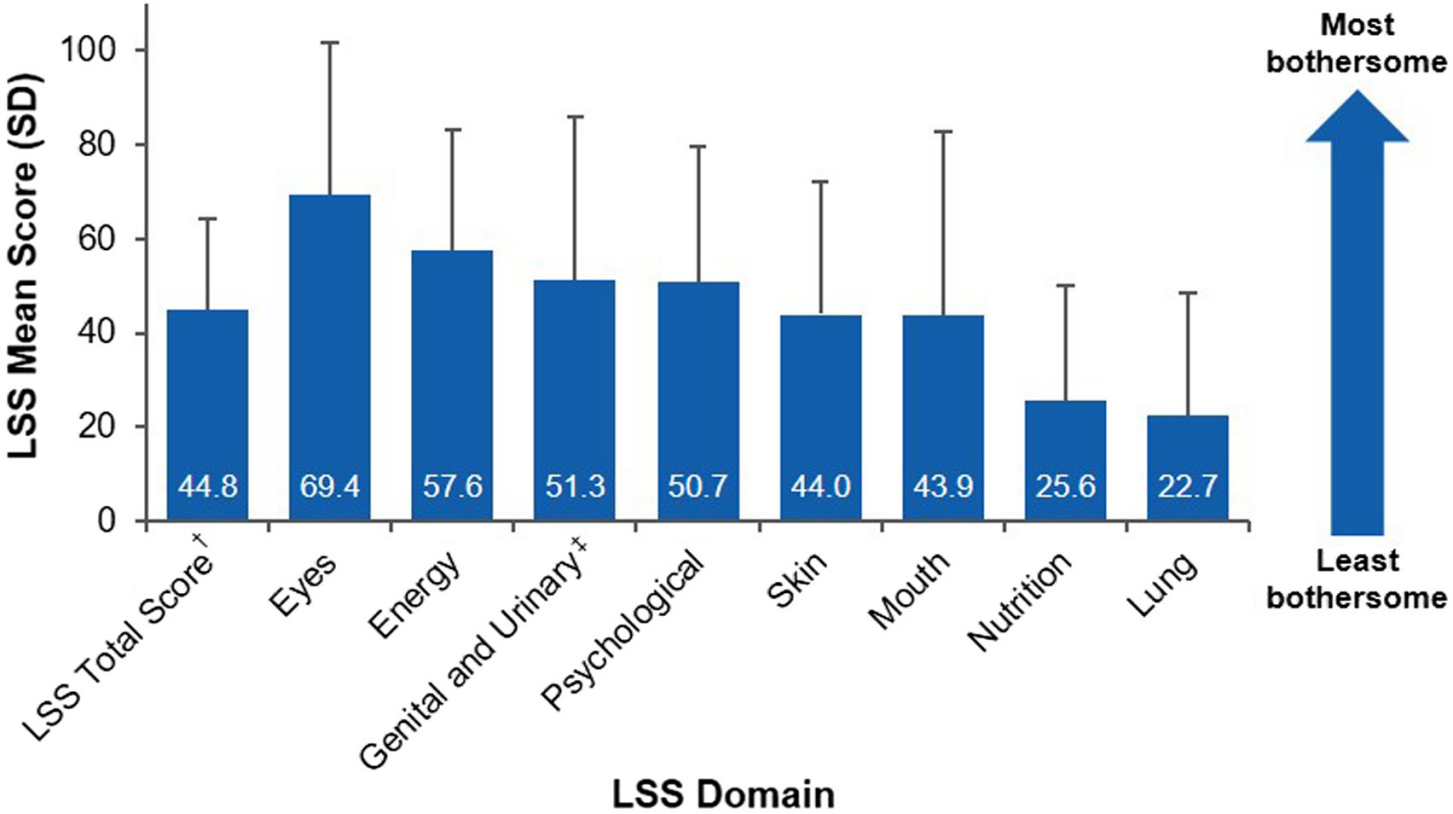
Total LSS summary score and subdomain scores when chronic GVHD symptoms were at their worst. GVHD, graft‐versus‐host disease; LSS, Lee Symptom Scale. ^†^Total score computed as the average of the 7 original subscale scores (excludes genital and urinary domain), as long as 4 or more subscales are available. ^‡^Captured using a separate subscale.

Survey participants recalled experiencing their most severe symptoms at a median (range) of 0.5 (0–19.2) years after chronic GVHD diagnosis, with symptoms remaining at their worst for a median (range) of 6.0 (0–242.0) months. For one‐third of respondents (*n* = 56), their most severe symptoms were reported to last for ≥1 year, with 9% (*n* = 15) of respondents experiencing their most severe symptoms for >5 years. Chronic GVHD severity at diagnosis was described as “moderate” or “severe” by most respondents (*n* = 71 [43.0%] and *n* = 57 [34.5%], respectively). When symptoms were at their worst, nearly all respondents described their chronic GVHD severity as moderate (*n* = 54 [32.7%]) or severe (*n* = 102 [61.8%]). Respondents reported a mean (SD; range) LSS total score of 44.8 (19.4; 2–100) when symptoms were at their worst. The highest mean (SD; range) LSS individual symptom scores were for the eyes (69.4 [32.4; 0–100]), followed by energy (57.6 [25.8; 0–100]) and psychological symptoms (50.7 [29.1; 0–100]) subscales (Figure 2). The subscale assessing genital and urinary domains also showed these symptoms to be bothersome (mean [SD], 51.3 [34.4]; range, 0–100).

**FIGURE 3:**
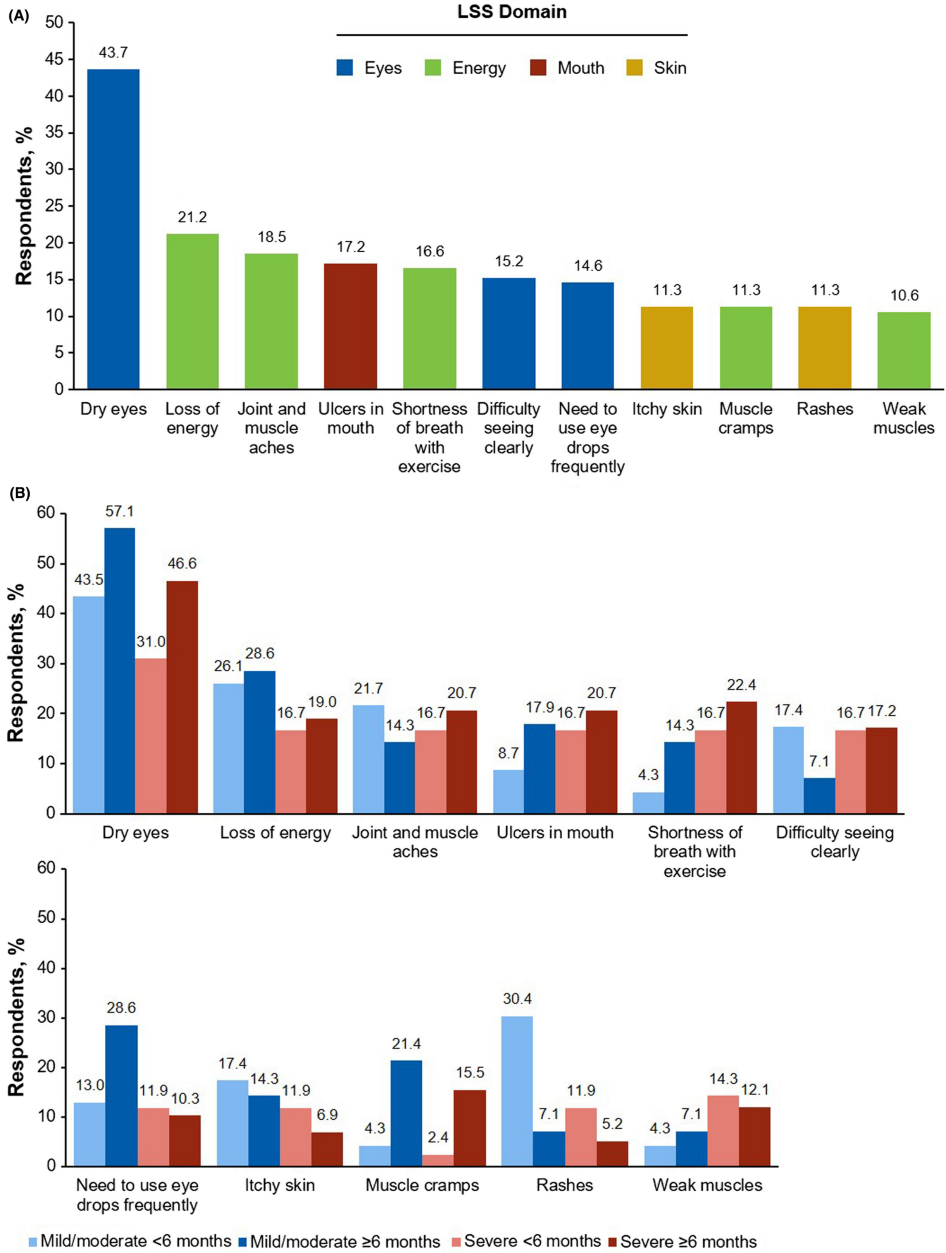
Respondent‐selected top 3 most bothersome chronic GVHD symptoms^†^ (A) overall and (B) by severity and length of their worst symptoms. Symptoms reported in ≥10% of patients overall are shown. GVHD, graft‐versus‐host disease; LSS, Lee Symptom Scale. ^†^Symptom categories were not mutually exclusive.

**FIGURE 4:**
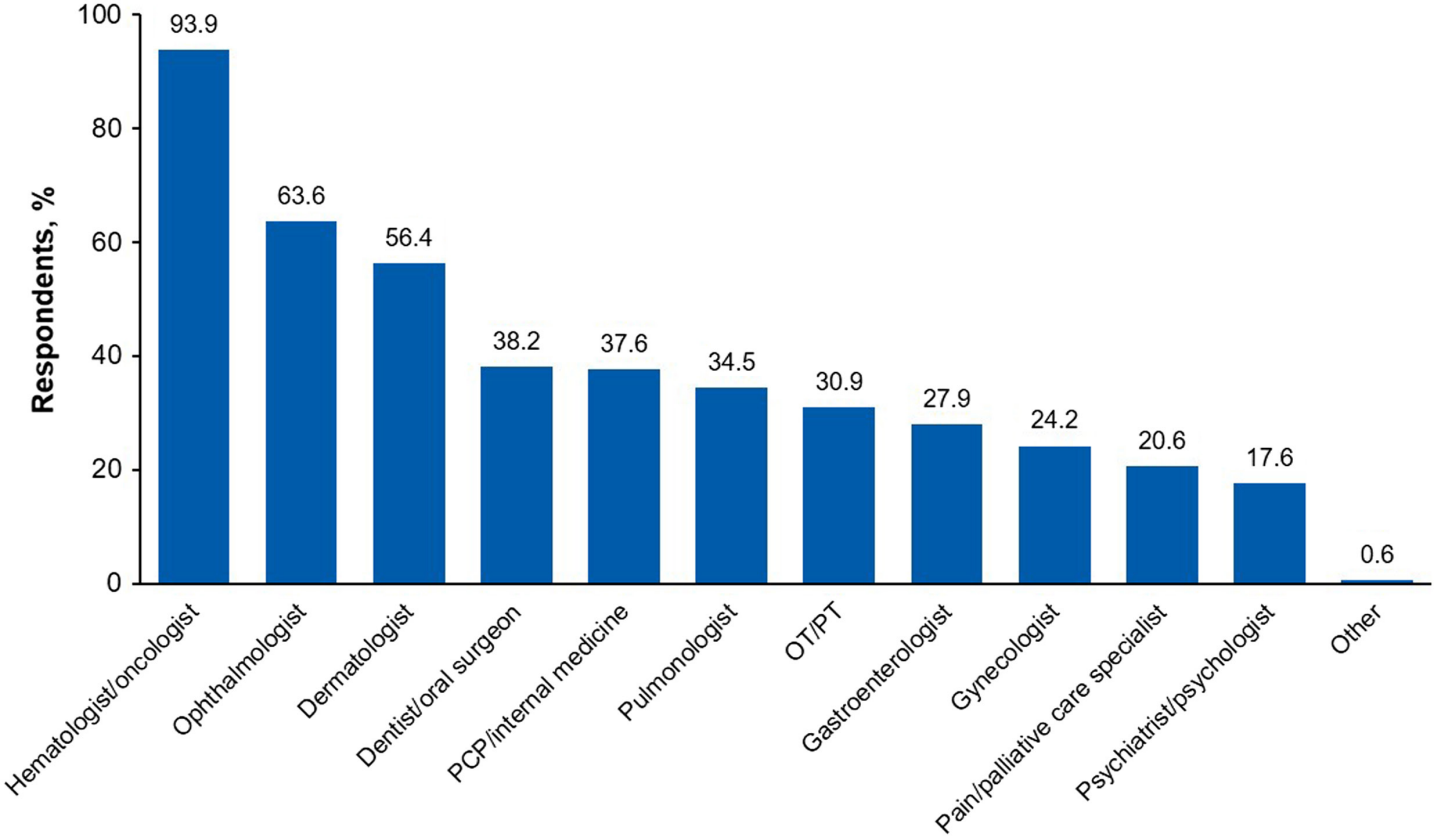
Types of specialists seen by survey respondents for their chronic GVHD. GVHD, graft‐versus‐host disease; OT, occupational therapist; PCP, primary care physician; PT, physical therapist.

Among participants who responded to a survey question regarding the “3 most bothersome symptoms” (*n* = 151), dry eyes was the most frequently selected response (*n* = 66 [43.7%]; Figure 3A). This was true irrespective of disease severity and duration, with the highest prevalence reported by respondents whose symptoms remained at their worst for ≥6 months (Figure 3B). Specifically among respondents reporting severe disease and symptoms lasting ≥6 months at their worst (*n* = 59), shortness of breath with exercise (*n* = 13 [22.4%]), joint/muscle aches (*n* = 12 [20.7%]), and ulcers in mouth (*n* = 12 [20.7%]) were also common and generally reported more frequently compared with patients with mild or moderate disease or those with shorter (ie, <6 months) disease duration (Figure 3B). Aside from hematologist/oncologist specialists (seen by 155 [93.9%] respondents), survey participants most frequently reported seeing ophthalmologists (*n* = 105 [63.6%]), dermatologists (*n* = 93 [56.4%]), and dentists/oral surgeons (*n* = 63 [38.2%]) for their chronic GVHD (Figure 4).

### 3.3 Effect of symptoms on HRQoL and daily activities

**FIGURE 5:**
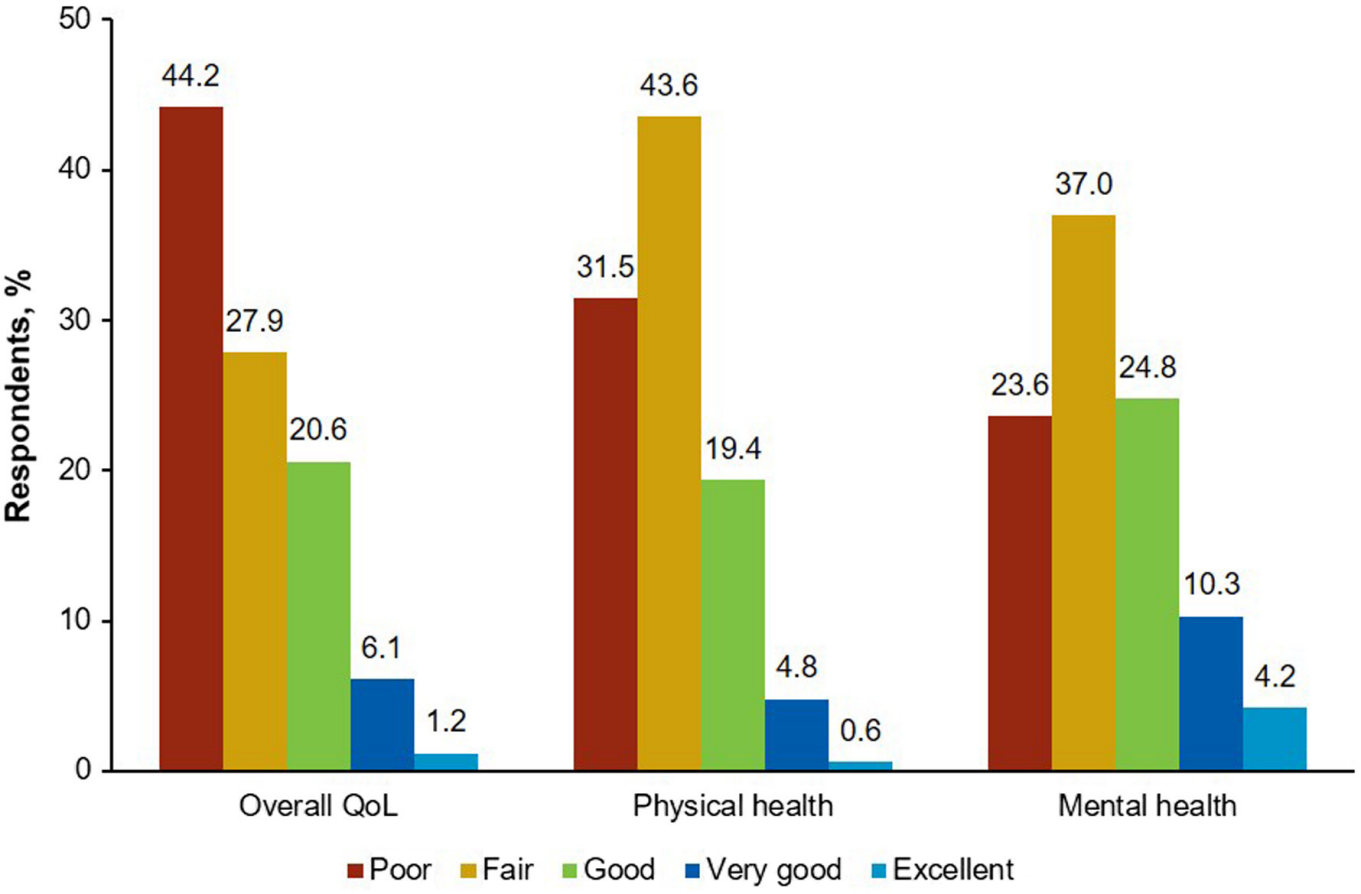
Respondent‐reported overall QoL, physical health, and mental health when chronic GVHD symptoms were at their worst. GVHD, graft‐versus‐host disease; QoL, quality of life.

**FIGURE 6:**
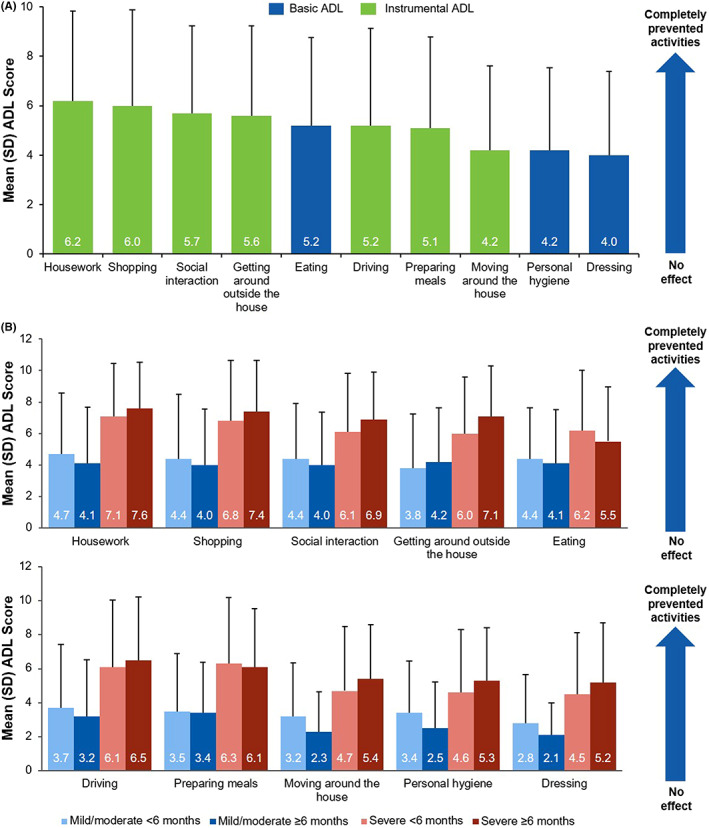
Impact of chronic GVHD on ADL^†^ when symptoms were at their worst (A) overall and (B) by symptom severity and duration. ADL, activities of daily living; GVHD, graft‐versus‐host disease. ^†^Coded score range: 1 (“no effect on my daily activities”) to 11 (“completely prevented me from doing my daily activities”). A higher score indicates greater impact on activities. Basic ADLs are skills required to manage basic physical needs; instrumental ADLs require more complex organizational and cognitive abilities. Activities with mean scores ≥4.0 are shown.

Out of possible options of “excellent,” “very good,” “good,” “fair,” and “poor,” nearly half (*n* = 73 [44.2%]) of respondents rated their overall HRQoL as poor when chronic GVHD symptoms were at their worst, with 52 (31.5%) reporting poor physical health and 39 (23.6%) reporting poor mental health (Figure 5). Further, only a small minority of respondents described their overall HRQoL (*n* = 12 [7.3%]), physical health (*n* = 9 [5.5%]), and mental health (*n* = 24 [14.5%]) as “very good” or “excellent” when symptoms were at their worst. When assessing the impact of chronic GVHD on activities of daily living on a coded scale of 1 (“chronic GVHD had no effect on my daily activities”) to 11 (“chronic GVHD completely prevented me from doing my daily activities”), respondents reported the highest scores (ie, most difficulty) with housework (mean [SD], 6.2 [3.6]; range, 1–11), shopping (6.0 [3.9]; 1–11), social interaction (5.7 [3.5]; 1–11), and getting around outside the house (5.6 [3.6]; 1–11; Figure 6A). However, it was also common for respondents to experience difficulty with basic activities of daily living, such as eating (mean [SD], 5.2 [3.6]; range, 1–11), personal hygiene (4.2 [3.3]; 1–11), and dressing (4.0 [3.4]; 1–11). Indeed, when chronic GVHD symptoms were at their worst, 41 (24.8%) respondents were unable to walk 50 feet without rest, and 113 (68.5%) were unable to walk 900 feet. Overall, survey participants with severe disease reported experiencing a greater impact on their daily activities compared with respondents with mild or moderate disease. In contrast, the perceived impact on daily activities was similar between respondents with shorter disease duration (<6 months) and those with longer disease duration (≥6 months) when stratified by disease severity (Figure 6B).

## 4 DISCUSSION

We describe results from an online survey assessing the symptom burden of chronic GVHD among adult patients in the United States and explore patients' perceptions of the effect of these symptoms on their HRQoL and daily activities. A geographically diverse sample of respondents recruited by consumer panels and patient advocacy groups completed the survey and was included in the analysis. A substantial proportion of survey participants reported protracted and severe chronic GVHD symptoms. The most burdensome patient‐reported symptoms were dry eyes, loss of energy, and joint/muscle aches. When asked to consider the period when chronic GVHD symptoms were at their worst, most survey respondents (62%) described their disease as severe, with the worst symptoms lasting for a median of 6 months. During that time period, nearly half of survey participants reported having poor HRQoL, and almost one‐third and one‐quarter of respondents described their physical and mental health, respectively, as poor. Survey respondents reported a large negative impact of their symptoms on daily activities, including basic activities of daily living such as eating, personal hygiene, and dressing. In particular, participants with self‐reported severe chronic GVHD generally had higher scores on activities of daily living, indicating greater impairment, compared with respondents with mild or moderate disease.

Several previous studies have examined the association of chronic GVHD symptoms with QoL. In a previous cross‐sectional natural history study of patients with chronic GVHD, disease severity was negatively associated with several outcome measures, including LSS, with the presence of sclerotic skin, joints/fascia manifestations, or lung involvement having the greatest detrimental effect overall, and gastrointestinal involvement having the greatest effect on LSS.^21^ Additionally, in a large prospective, longitudinal study of patients with chronic GVHD, those with joint and fascia manifestations had lower QoL scores compared with patients without joint or fascia involvement.^24^ A previous longitudinal, observational study showed that more than half of patients (57%) with chronic GVHD experienced ocular symptoms within 2 years of diagnosis, and that these patients had worse scores on the Functional Assessment of Cancer Therapy Bone Marrow Transplantation compared with patients without ocular GVHD.^25^ Interestingly, in the current study, survey participants reported that the most bothersome effects were related to eye symptoms (eg, dry eyes), suggesting that this cohort may represent a somewhat healthier patient population than the chronic GVHD population overall. Alternatively, it is possible that the design or duration of this online survey may have made respondents more mindful of their eye symptoms in particular. Of note, it is not possible to entirely rule out the potential for contributing effects of other factors (eg, concomitant conditions, cancer‐related or other therapies) on eye symptoms in this survey study.

Several studies have previously reported psychological distress among patients with chronic GVHD,^16^, ^23^, ^26^, ^27^ and the physical symptoms of chronic GVHD may be substantial contributors to mental health issues. In a prospective observational cohort study from the Chronic GVHD Consortium, patients with self‐reported depression and anxiety symptoms (per the LSS psychological subscale) had higher overall chronic GVHD symptom burden and worse QoL compared with patients without depression or anxiety symptoms.^23^ Furthermore, patients with self‐reported depression symptoms had worse overall survival versus those without. In the present study, high scores were observed in the psychological domain of the LSS, indicating greater impairment, and many patients reported poor mental health. However, relatively few survey respondents received care from a psychiatrist or psychologist for their chronic GVHD, indicating a potential opportunity to improve patient care through addressing mental health issues. Finally, multiple studies have reported worse QoL among patients with active versus resolved chronic GVHD,^13^, ^16^, ^28^ suggesting that effective treatment of chronic GVHD and management of associated symptoms could reverse the detrimental effects on HRQoL.

The study was limited by the potential for selection bias regarding who completed the survey, as recruitment was limited to patient advocacy groups and consumer panels, so types of respondents may not reflect the chronic GVHD population at large. Additionally, patients with the most severe manifestations of chronic GVHD may have been too ill to participate in this cross‐sectional survey. Based on recruitment methodology, it is not possible to determine how many patients received the initial invitation and decided whether or not to access the survey. The survey was only available online and limited to those who could read and understand English. This was also a cross‐sectional survey and reporting of specific dates, disease severity, symptoms, and the effects of those symptoms may have been subject to recall bias. For example, respondents may have had difficulty recalling specific dates (eg, date of transplant and date of chronic GVHD diagnosis) and provided similar responses for each, potentially skewing the data to appear as though chronic GVHD diagnosis occurred immediately upon transplant. This example also reflects the potential that patients do not fully understand the disease course or may not be able to differentiate a previous experience with acute GVHD from their chronic GVHD. To better ascertain the full burden of living with chronic GVHD, patients were asked to reflect on the time when symptoms were at their worst while answering questions, rather than just at the time of the survey, which may have further increased the potential for recall bias. As many patients with chronic GVHD experience precedent acute GVHD in the real‐world setting, respondents who previously had acute GVHD were more prone to the recall bias. Additional study limitations include that chronic GVHD diagnoses and clinical characteristics (eg, disease duration and severity) were self‐reported by survey respondents and were not confirmed by a clinician. Furthermore, details regarding specific treatments received for chronic GVHD, including toxicities experienced and related impact on symptoms, were not collected. Use of a cross‐sectional versus longitudinal study design did not allow for assessment of changes in chronic GVHD symptom burden and subsequent impact on QoL and activities over time. Finally, the survey evaluated the overall burden of chronic GVHD symptoms and was not designed to capture or formally evaluate associations between the effects of specific symptoms on outcome measures.

In conclusion, in an online survey assessing the impact of chronic GVHD symptoms on HRQoL and daily activities, respondents reported that their symptoms severely interfered with their physical function and ability to perform both basic and instrumental activities of daily living. These findings further highlight the need for effective therapies that alleviate symptoms of chronic GVHD and improve patients' physical and social function among long‐term HSCT survivors.

## AUTHOR CONTRIBUTIONS

Jingbo Yu: conceptualization, formal analysis, and writing – review and editing. Betty K. Hamilton: conceptualization, formal analysis, and writing – review and editing. James Turnbull: conceptualization, formal analysis, and writing – review and editing. Susan K. Stewart: conceptualization, formal analysis, and writing – review and editing. Alla Vernaya: conceptualization, formal analysis, and writing – review and editing. Valkal Bhatt: conceptualization, formal analysis, and writing – review and editing. Oren Meyers: conceptualization, methodology, project administration, and writing – review and editing. John Galvin: conceptualization, formal analysis, and writing – review and editing.

## CONFLICT OF INTEREST

JY, VB, and JG are employees and shareholders of Incyte Corporation. BKH has served on an advisory board for Equilium and Syndax Pharmaceuticals. JT, AV, and OM are employees of IQVIA, the company commissioned by Incyte Corporation to conduct this study. SKS's not‐for‐profit employer has received unrestricted educational grants in the past 3 years from Incyte Corporation, Kadmon, and Pharmacyclics.

## ETHICS APPROVAL STATEMENT

The study was conducted in accordance with the ethical principles embodied by the Declaration of Helsinki and Good Clinical Practice. The survey, including revisions based on the pilot in 3 participants, was reviewed and approved by the New England Institutional Review Board before implementation.

## CLINICAL TRIAL REGISTRATION

Not applicable; the study does not report on a clinical trial.

## PATIENT CONSENT STATEMENT

All survey respondents provided informed consent via an online consent form before screening and survey completion.

## PERMISSION TO REPRODUCE MATERIALS FROM OTHER SOURCES

Not applicable.

## Supporting information


Table S1

## Data Availability

Access to individual patient‐level data is not available for this study. Information on Incyte's clinical trial data sharing policy and instructions for submitting clinical trial data requests are available at: https://www.incyte.com/Portals/0/Assets/Compliance%20and%20Transparency/clinical‐trial‐data‐sharing.pdf?ver=2020‐05‐21‐132838‐960
