# Supplementary material for: Patient‐reported symptom burden and impact on daily activities in chronic graft‐versus‐host disease
Source: Cancer Med. 2022 Nov 16;12(3):3623–33. doi: 10.1002/cam4.5209 (PMC9939096; doi:10.1002/cam4.5209)
Supplement: Supplementary file 1 — Table S1 [file CAM4-12-3623-s001.pdf]

Patient-reported symptom burden and effects on daily activities in chronic graft-versus-host disease

Jingbo Yu, MD, PhD,[1]* Betty K. Hamilton, MD,[2]* James Turnbull,[3] Susan K. Stewart,[4] Alla Vernaya,[3] Valkal Bhatt, PharmD,[1] Oren Meyers, PhD,[3] John Galvin, MD, MPH[1]

[1]Incyte Corporation, Wilmington, DE, USA; [2]Cleveland Clinic, Cleveland, OH, USA; [3]Patient Centered Endpoints, IQVIA, New York, NY, USA; [4]Blood & Marrow Transplant Information Network, Highland Park, IL, USA

* Dr. Yu and Dr. Hamilton should be considered joint first author.

SUPPORTING INFORMATION

Contents

Supplemental Table S1. Symptoms assessed by LSS domain 2

Supplemental Table S1. Symptoms assessed by LSS domain

LSS domain	Individual symptoms
Eyes	• Dry eyes<br>• Need to use eye drops frequently<br>• Difficulty seeing clearly
Energy	• Shortness of breath with exercise<br>• Joint and muscle aches<br>• Limited joint movement<br>• Muscle cramps<br>• Weak muscles<br>• Loss of energy<br>• Need to sleep more/take naps
Psychological	• Depression<br>• Anxiety<br>• Difficulty sleeping
Skin	• Abnormal skin color<br>• Rashes<br>• Thickened skin<br>• Sores on skin<br>• Itchy skin
Mouth	• Need to avoid certain foods due to mouth pain<br>• Ulcers in mouth
Nutrition	• Receiving nutrition from an IV line or feeding tube<br>• Difficulty swallowing solid foods<br>• Difficulty swallowing liquids<br>• Vomiting<br>• Weight loss
Lung	• Frequent cough<br>• Colored sputum<br>• Shortness of breath at rest<br>• Need to use oxygen<br>• Fevers
Genital and urinary†	• Vaginal discomfort<br>• Vaginal dryness<br>• Vaginal irritation<br>• Lack of sex drive

IV, intravenous; LSS, Lee Symptom Scale.
† Genital and urinary symptoms are captured by a separate subscale and are not included in the LSS total score.
